# Suitability of sentinel abattoirs for syndromic surveillance using provincially inspected bovine abattoir condemnation data

**DOI:** 10.1186/s12917-015-0349-1

**Published:** 2015-02-15

**Authors:** Gillian D Alton, David L Pearl, Ken G Bateman, W Bruce McNab, Olaf Berke

**Affiliations:** Department of Population Medicine, Ontario Veterinary College, University of Guelph, Guelph, ON N1G 2 W1 Canada; Ontario Ministry of Agriculture & Food, Guelph, ON N1G 4Y2 Canada; Department of Mathematics and Statistics, University of Guelph, Guelph, ON N1G 2 W1 Canada

## Abstract

**Background:**

Sentinel surveillance has previously been used to monitor and identify disease outbreaks in both human and animal contexts. Three approaches for the selection of sentinel sites are proposed and evaluated regarding their ability to capture overall respiratory disease trends using provincial abattoir condemnation data from all abattoirs open throughout the study for use in a sentinel syndromic surveillance system.

**Results:**

All three sentinel selection criteria approaches resulted in the identification of sentinel abattoirs that captured overall temporal trends in condemnation rates similar to those reported by the full set of abattoirs. However, all selection approaches tended to overestimate the condemnation rates of the full dataset by 1.4 to as high as 3.8 times for cows, heifers and steers. Given the results, the selection approach using abattoirs open all weeks had the closest approximation of temporal trends when compared to the full set of abattoirs.

**Conclusions:**

Sentinel abattoirs show promise for integration into a food animal syndromic surveillance system using Ontario provincial abattoir condemnation data. While all selection approaches tended to overestimate the condemnation rates of the full dataset to some degree, the abattoirs open all weeks selection approach appeared to best capture the overall seasonal and temporal trends of the full dataset and would be the most suitable approach for sentinel abattoir selection.

## Background

There are various approaches to conduct disease surveillance including sentinel and syndromic surveillance. Sentinel surveillance is a form of surveillance which involves a limited number of recruited participants or organizations, such as farms, veterinarians, abattoirs, healthcare providers or hospitals, which report on certain health events to give an indication of what may be happening in the general population [1]. Sentinel surveillance is a strategy used to sample timely data in a relatively inexpensive manner rather than collect information on the general population, when population-based data collection is unfeasible in a timely or cost-effective manner [2]. Syndromic surveillance involves the amalgamation of signs/symptoms using data from non-traditional data sources [3]; the signs/symptoms are grouped into classifications called ‘syndromes’ and are used to track disease trends in populations and signal a possible outbreak that warrants further investigation [3].

Sentinel surveillance has been previously used in both human and animal health settings for a variety of disease outcomes. Sentinel surveillance has been used to monitor or identify outbreaks of infectious diseases and to monitor the activity of certain health conditions which can change due to environmental conditions. Though used less often in animal health applications than in human health, sentinel surveillance has been used successfully for surveillance in various applications. For example, following the emergence of Bluetongue virus serotype 8 in Central Europe in 2006, causing a large scale outbreak in 2007 in several countries in Europe, a Bluetongue sentinel surveillance program was established in Belgium in 2010. This surveillance program was intended to demonstrate the absence of Bluetongue virus [4]. This program randomly selected a total of 300 dairy herds, with 30 herds selected from each of the Belgian provinces. The criteria for selection of herds was based on dairy herds that were expected to have a minimum of 15 animals present between 4 and 12 months of age at the start of the sentinel program [5]. Other studies have combined both sentinel and syndromic surveillance and utilize data from sentinel veterinarians or veterinary practices [6,7].

Animal health data including abattoir condemnation data have emerged recently as a novel data stream for syndromic surveillance of diseases of animal and public health importance [6,8-13]. Ontario provincial abattoir data have been recently explored as a potential source of information for food animal syndromic surveillance [8-10]. Data from Ontario provincial abattoirs are particularly appealing as they can provide a more regionally specific picture of emerging diseases in Ontario, as the cattle shipped to these abattoirs originate from farms from approximately a 100 km radius [9]. However, there are approximately 100–150 provincial abattoirs in Ontario, many of which are open sporadically and have differing processing capacities, which may bias the results of quantitative methods for disease surveillance. It may be beneficial to conduct enhanced and targeted surveillance at select abattoirs in order to gather more accurate data for syndromic surveillance. In addition, by selecting specific sentinel abattoirs for inclusion in a sentinel syndromic surveillance system, it would allow for more intensive and specialized training of inspectors for syndromic surveillance. Furthermore, if sentinel abattoirs were selected properly, they could help to reduce the cost of surveillance while still being representative of the overall condemnation trends in all Ontario provincial abattoirs. Cost efficiency could result from limiting the number of abattoirs requiring investigation during potential aberrations in condemnation rate, and by reducing the number of abattoirs requiring targeted surveillance and specialized training of inspectors. The criteria used to select sentinel abattoirs require consideration. We proposed three sentinel site selection approaches and compared their ability to detect respiratory disease trends in bovine abattoir condemnation data for use in a sentinel-based syndromic surveillance system.

Pneumonic lung condemnation rates from all Ontario abattoirs processing cattle throughout the 2001–2007 period were compared to those collected from sentinel sites based on three selection approaches: (1) abattoirs processing cattle all weeks of the year; (2) abattoirs processing at least 6500 cattle per year (based on data from abattoirs in the upper 95^th^ percentile in processing capacity); and (3) multi-criteria selection approach of abattoirs that met a predefined set of criteria related to abattoir processing capacity, and animal classes represented.

The goal of the study was to compare abattoir selection approaches for a sentinel surveillance system based on provincially inspected abattoirs in Ontario. Specific objectives of this study were the following: (1) determine the suitability of sentinel abattoirs for food animal syndromic surveillance using provincial abattoir pneumonic lung condemnation data as an example; and (2) determine which design is most efficient and representative for pneumonic lung condemnation rate in terms of spatial distribution, temporal trends and relative differences between animal classes when compared to data from all the abattoirs over the study period.

## Methods

### Data source

Pneumonic lung portion condemnation data were obtained from the Food Safety Decision Support System (FSDSS) database maintained by the Ontario Ministry of Agriculture, Food and Rural Affairs (OMAFRA). The database contains information regarding the number and reason for daily organs/body systems condemnations in provincially inspected abattoirs in Ontario. The condemnation category of pneumonic lung was selected for these analyses, as this category represents a major health issue for beef cattle and was among the most frequently reported portion condemnations by provincial inspectors during the study period. Pneumonic lung condemnation refers to bovine lungs which were condemned for lesions indicative of a previous localized and resolved antero-ventral pneumonia infection (personal communication Abdul Rehmtulla, DVM, OMAFRA, Stone Road, Guelph, Ontario).

Data were extracted from the Food Safety Decision Support System (FSDSS) database for cattle animal classes: bulls, calves, cows, heifers, and steers from January 1, 2001 to December 31, 2007. Within the FSDSS database, calves are defined as any animal under 396 lbs dressed weight. All other animal classes are classified at the discretion of the inspector based on age, weight, breed and sex (personal communication Alexandra Reid, DVM, PhD, OMAFRA, Stone Road, Guelph, Ontario). Missing geographical coordinates for abattoirs were approximated using postal codes and/or addresses with the address geocoding software GeoPinpoint Suite 6.4 (DMTI Spatial Inc., Markham, Ontario, Canada). Using the FSDSS database, further variables were created for each month: geographical coordinates of abattoir, year, season, number of weeks an abattoir was operating each year, total number of pneumonic lung condemnations, total number of cattle processed each year, and animal class. Animal class included five categories: bulls, cows, calves, heifers, and steers. Bulls were excluded from subsequent analyses due to missing data and inconsistencies in the use of this classification. The number of weeks an abattoir was operating each year was determined by the total number of weeks in which at least one bovine animal was processed. The total number of animals processed each year was calculated from the total number of condemned cattle plus the number of cattle fit for consumption. The agricultural region where an abattoir was located was classified as: central, eastern, northern, southern or western Ontario using the Ontario Census Agricultural Region boundaries (Statistics Canada, Census Agricultural Regions, Census year 2001). The regional location of each abattoir was determined using the point-in-polygon technique with geographic information system software ArcGIS 9.2 (ESRI, Redlands, California, USA).

This study conducted statistical analyses utilizing a pre-existing government database. As no experimentation or use confidential information for people or animals was used in this study, no ethical approval application was needed.

### Descriptive analyses

Monthly bovine pneumonic lung condemnation rates were calculated using data from all abattoirs slaughtering cattle during the study period of January 1, 2001 – December 31, 2007. Condemnation rates were calculated by dividing the total number of pairs of lungs condemned under the pneumonic lung classification each month by the total number of slaughtered bovines for each animal class (e.g., calves, cows, heifers and steers).

During the study period, the number of abattoirs in operation varied from year to year, which was likely due to economic and regulatory changes in the cattle industry. There were a total of 211 provincial abattoirs slaughtering a total of 1,155,535 cattle from 2001–2007. The monthly cattle slaughtered per animal class have been reported in Alton et al. [8]. During the study period there were 36,883 lungs condemned representing approximately 9% of all portion condemnation and among the most frequently reported condemnation in cattle. Further discussion of these data can be found in a previous study by Alton et al. [8]. However, there were only 98 abattoirs that remained in operation for the entire study period and were therefore used to represent the full set of abattoirs in this study. This number is consistent with the current number of abattoirs processing cattle in Ontario in 2014 [14]. There were a total of 33,182 lungs condemned and 856,467 cattle processed during the study period at these 98 abattoirs. Three different design approaches for a sentinel syndromic surveillance system were compared to all data from the full set of 98 abattoirs. The first sentinel selection approach, which we refer to as abattoirs open all weeks, uses data from abattoirs processing cattle 52 weeks per year. The second approach, which we refer to as large abattoirs; uses data from abattoirs in the upper 95^th^ percentile in processing capacity (processing at least 6500 cattle per year). The third approach, which we refer to as multi-criteria approach, uses data from abattoirs that met the following criteria for each year of the study period: processed at least 499 cattle per year (representing the median total number of cattle processed based on data from all 211 abattoirs open from 2001 – 2007), processed at least 1 bovine carcass 44 weeks or more each year (representing the median number of weeks cattle were processed based on data from all 211 abattoirs open from 2001–2007), and processed cattle representing all animal classes (calves, cows, heifers, and steers).

Data obtained using the three design approaches were summarized graphically using the raw monthly condemnation rates of each animal class. In addition, data were summarized in terms of the number of abattoirs included in each selection approach, percentage of shared abattoirs between each selection approach, and geographical representativeness of each selection approach according to the distribution of abattoirs among census agricultural regions in Ontario.

### Statistical analyses

To evaluate the overall condemnation rates within each animal class, a univariable negative binomial model was used to compare monthly pneumonic lung condemnation rates from all sentinel site selection approaches and the full set of abattoirs using a categorical variable for each of the 3 sentinel design approaches. To determine whether the overall seasonal and temporal trends of the full dataset were being captured within each sentinel selection approach, separate multi-level negative binomial regression models were used with a random intercept for abattoir using the xtnbreg command in Stata. This model allows the random effect to follow a beta distribution and for the overdispersion parameter to vary by abattoir. The negative binomial model was used to evaluate the association of monthly condemnation rates of pneumonic lungs for each sentinel selection approach with year, season and animal class. All covariates were evaluated for statistical significance individually and then in a multivariable multi-level negative binomial model. All covariates were forced into the models regardless of univariable significance, due to the identification of these variables as important predictors for pneumonic lung condemnation rates in a previous study [8] and to evaluate overall temporal trends in the data. All statistical models include the log of the number of animals slaughtered for a specific animal class in the offset.

For all regression models, the decision to use a negative binomial model instead of a Poisson regression model was based on evaluating the Akaike Information Criterion (AIC) value of both models and the significance of the over-dispersion term of the negative binomial model. The offset of the negative binomial model was the natural log of the total number of slaughtered cattle for the abattoirs included for each sentinel site selection approach for each animal class and month-year period during the study period. All statistical analyses were conducted using Stata 12 (Stata Corp., College Station, Texas, USA).

## Results

### Descriptive statistics

Three approaches for sentinel abattoir selection were compared to pneumonic lung condemnation rates for each animal class from the full set of abattoirs (Table 1). Abattoirs for each sentinel selection approach were chosen from a total of 98 abattoirs processing cattle for the entire study period. The number of abattoirs selected for each sentinel selection approach varied from 7 to 45 (Table 1). In assessing the percentage of overlap of the selected abattoirs among the sentinel site selection approaches, the percentage of shared abattoirs ranged from approximately 7% to 71%, with abattoirs open all weeks and multi-selection criteria approaches having the highest percentage of abattoirs in common (Table 2). All selection approaches led to surveillance systems based on sentinel abattoir condemnation rates representing all census agricultural regions across Ontario with the exception of using the large abattoir criterion, which did not include abattoirs from eastern and northern Ontario (Table 3).

**Table 1:** **Summary of sentinel abattoirs selection approaches for Ontario provincial abattoirs (2001 – 2007)**

**Sentinel site selection approach**	**Selection description**	**Number of abattoirs**
**Full dataset**	Abattoirs processing at least 1 bovine carcass each year of study period (2001 – 2007)	98
**Abattoirs open all weeks**	Abattoirs processing at least 1 bovine carcass 52 weeks per year	45
**Multi-criteria**	Selection of abattoirs meeting the following criteria: processed at least 499 cattle per year, processed at least 1 bovine carcass 44 weeks or more per year, processed cattle representing all animal classes (calves, cows, heifers and steers)	44
**Large abattoirs**	Abattoirs processing ≥ 6500 cattle per year	7

**Table 2:** **Percentage of shared abattoirs between sentinel selection approaches**

**Selection approaches**	**Full dataset** ^**1**^ **(N = 98)**	**Abattoirs open all weeks** ^**2**^ **(N = 45)**	**Selection criteria** ^**3**^ **(N = 44)**	**Large abattoirs** ^**4**^ **(N = 7)**
**Full dataset** ^**1**^ **(N = 98)**	100% (98)	45.9% (45)	44.9% (44)	7.1% (7)
**Abattoirs open all weeks** ^**2**^ **(N = 45)**	45.9% (45)	100% (45)	71.1% (32)	15.6% (7)
**Selection criteria** ^**3**^ **(N = 44)**	44.9% (44)	71.1% (32)	100% (44)	6.8% (3)
**Large abattoirs** ^**4**^ **(N = 7)**	7.1% (7)	15.6% (7)	6.8% (3)	100% (7)

^1^Abattoirs processing at least 1 bovine carcass each year of study period (2001 – 2007).

^2^Abattoirs processing at least 1 bovine carcass 52 weeks per year.

^3^Selection of abattoirs meeting the following criteria: processed at least 499 cattle per year, processed at least 1 bovine carcass 44 weeks or more per year, processed cattle representing all animal classes (calves, cows, heifers and steers).

^4^Abattoirs processing ≥ 6500 cattle per year.

**Table 3:** **Distribution of provincial abattoirs 2001 – 2007 among census agricultural regions in Ontario based on three sentinel selection approaches**

**Selection approach**	**Number of abattoirs**	**Central Ontario**	**Eastern Ontario**	**Northern Ontario**	**Southern Ontario**	**Western Ontario**
**Full dataset** ^**1**^	98	18% (18)	15% (15)	7% (7)	36% (35)	23% (23)
**Abattoirs open all weeks** ^**2**^	45	18% (8)	13% (6)	7% (3)	38% (17)	24% (11)
**Multi-criteria** ^**3**^	44	9% (4)	18% (8)	5% (2)	39% (17)	30% (13)
**Large abattoirs** ^**4**^	7	29% (2)	0% (0)	0% (0)	43% (3)	29% (2)

^1^Abattoirs processing at least 1 bovine carcass each year of study period (2001 – 2007).

^2^Abattoirs processing at least 1 bovine carcass 52 weeks per year.

^3^Selection of abattoirs meeting the following criteria: processed at least 499 cattle per year, processed at least 1 bovine carcass 44 weeks or more per year, processed cattle representing all animal classes (calves, cows, heifers and steers).

^4^Abattoirs processing ≥ 6500 cattle per year.

**Figure 1:**
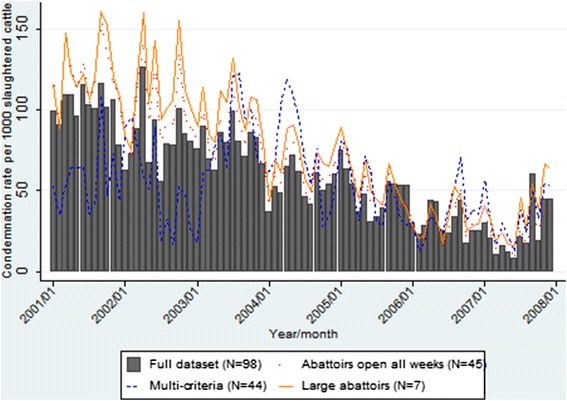
**Comparison of pneumonic lung condemnation rates in calves for sentinel site selection approaches and full dataset 2001–2007.**

**Figure 2:**
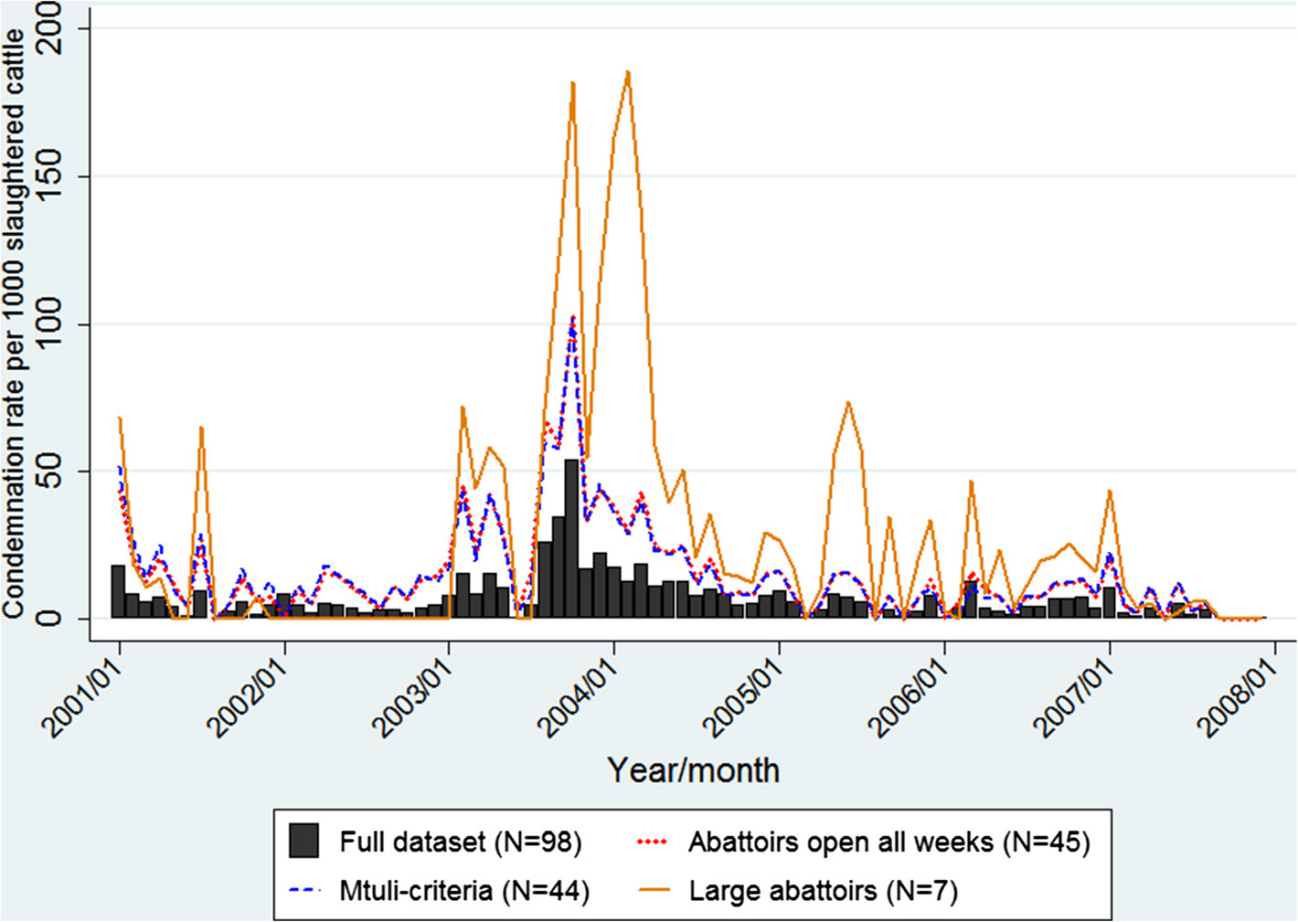
**Comparison of pneumonic lung condemnation rates in cows for sentinel site selection approaches and full dataset 2001 – 2007.**

**Figure 3:**
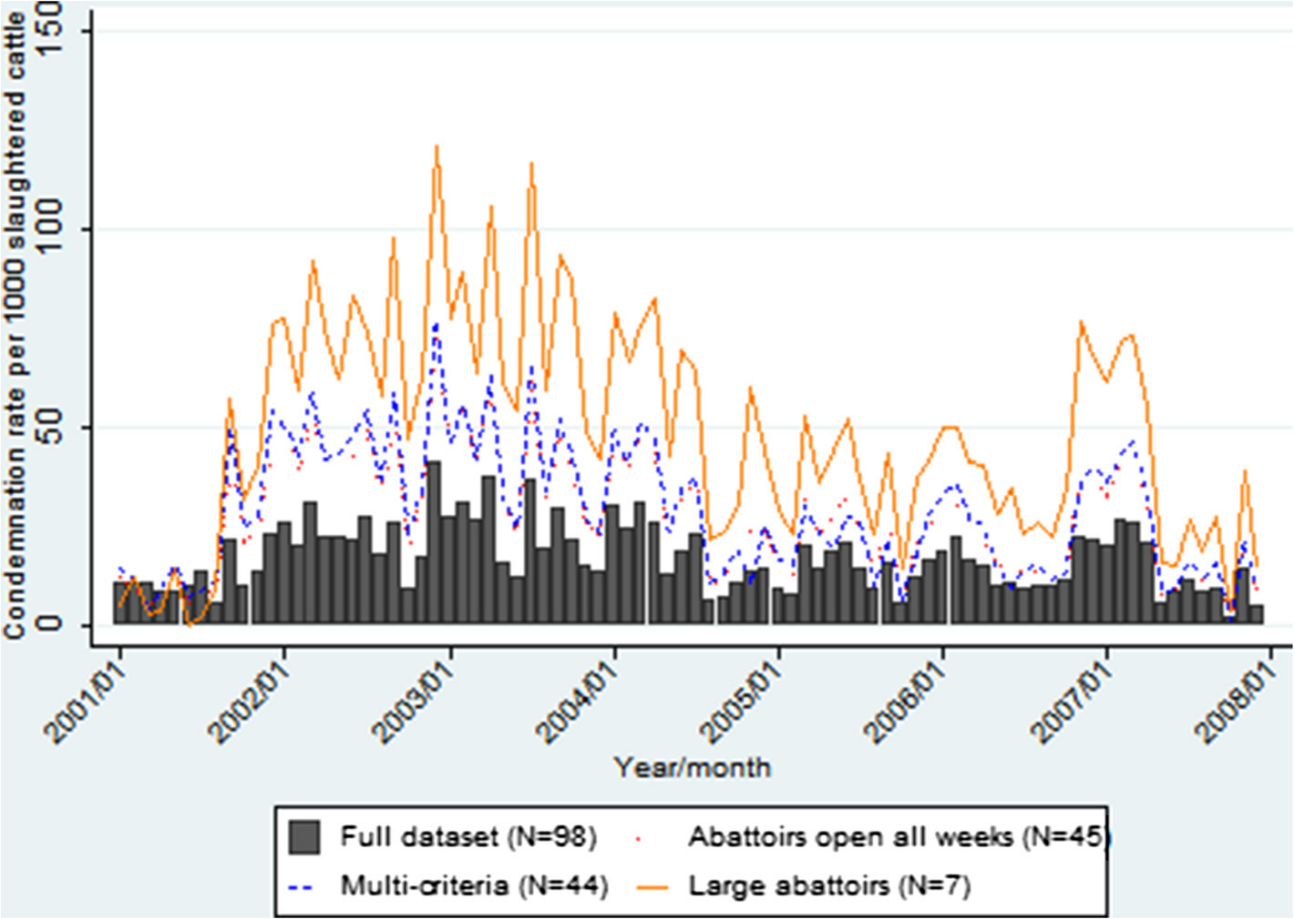
**Comparison of pneumonic lung condemnation rates in heifers for sentinel site selection approaches and full dataset 2001–2007.**

**Figure 4:**
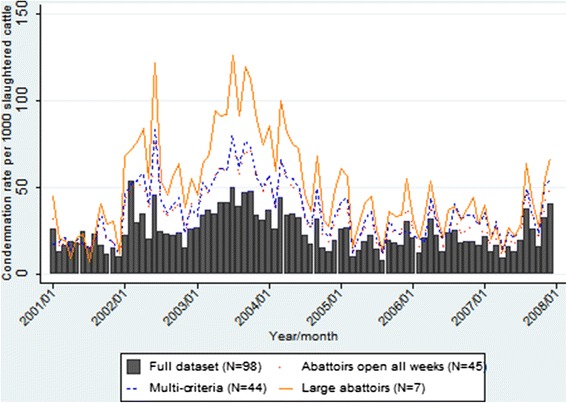
**Comparison of pneumonic lung condemnation rates in steers for sentinel site selection approaches and full dataset 2001–2007.**

The temporal condemnation rate graphs for all data indicate a gradual decrease in pneumonic lung condemnation rates over time in calves from approximately 100 condemnations per 1000 slaughtered calves in 2001 to approximately 20 condemnations per 1000 slaughtered calves in 2007 (Figure 1). In comparison, pneumonic lung condemnation rates in cows, heifers and steers remained much more stable over the study period (Figures 2, 3 and 4). The overall monthly condemnation rates for the full set of abattoirs compared to the 3 sentinel site selection approaches for each animal class had similar distributions for calves, heifers and steers (Figure 1, 3 and 4), with the exception of the large abattoir selection approach, which tended to have more variability when compared to the other selection approaches and full set of abattoirs.

Based on descriptive plots for calves (Figure 1), the multi-criteria selection approach did not show a good fit with the full dataset, with alternating trends of either overestimating or underestimating the condemnation rate of the full dataset. However, the other two alternative selection approaches tended to approximate the overall secular trends of the full dataset, but generally overestimated the condemnation rates of the full dataset throughout the study period. Based on the descriptive plots for cows, heifers and steers (Figures 2, 3 and 4), all three design approaches tended to overestimate the condemnation rate relative to the full set of data, however, the large abattoir selection approach had the largest overestimation in all animal classes.

### Negative binomial models

We used a negative binomial regression model to compare the monthly pneumonic lung condemnation rates from the 3 sentinel surveillance system selection approaches for each animal class to the full dataset. The model involving calves (Table 4a) found a difference between the large abattoir selection approach and the full set of data. In comparison, negative binomial modelling of cow data (Table 4b) found that all 3 design approaches overestimated the condemnation rate by a factor of 2 to 4 times, when compared to the full dataset. Similarly, relative to the full dataset, all the sentinel selection approaches overestimated the condemnation rates for heifers (Table 4c) and steers (Table 4d).

**Table 4:** **Negative binomial regression models comparing monthly pneumonic lung condemnation rates for all abattoirs for each animal class with three methods of sentinel abattoir selection**

**Sentinel selection method**	**IRR**	**95% CI**	**P-value**
**a) Calves**			
Full dataset^1^	---	---	---
Abattoirs open all weeks^2^	1.12	0.95 – 1.32	0.16
Multi-criteria^3^	0.90	0.76 – 1.06	0.21
Large abattoirs^4^	1.25	1.06 – 1.47	0.01
**b) Cows**			
Full dataset^1^	---	---	---
Abattoirs open all weeks^2^	2.08	1.49 – 2.91	<0.01
Multi-criteria^3^	2.06	1.47 – 2.89	<0.01
Large abattoirs^4^	3.83	2.73 – 5.38	<0.01
**c) Heifers**			
Full dataset^1^	---	---	---
Abattoirs open all weeks^2^	1.61	1.35 – 1.94	<0.01
Multi-criteria^3^	1.70	1.42 – 2.04	<0.01
Large abattoirs^4^	2.86	2.38 – 3.43	<0.01
**d) Steers**			
Full dataset^1^	---	---	---
Abattoirs open all weeks^2^	1.39	1.20 – 1.61	<0.01
Multi-criteria^3^	1.48	1.28 – 1.72	<0.01
Large abattoirs^4^	2.02	1.74 – 2.34	<0.01

^1^Abattoirs processing at least 1 bovine carcass each year of study period (2001 – 2007).

^2^Abattoirs processing at least 1 bovine carcass 52 weeks per year.

^3^Selection of abattoirs meeting the following criteria: processed at least 499 cattle per year, processed at least 1 bovine carcass 44 weeks or more per year, processed cattle representing all animal classes (calves, cows, heifers and steers).

^4^Abattoirs processing ≥ 6500 cattle per year.

There were similar seasonal and temporal trends noted among all sentinel site selection approaches and the full set of abattoirs (Table 5a – d). However, the abattoirs open all weeks sentinel selection approach tended to have coefficients and trends which best resembled the seasonal and temporal trends of the full dataset. Abattoirs open all weeks also tended to have the closest approximation of coefficients when compared to the full dataset for the animal class variable.

**Table 5:** **Multivariable multilevel negative binomial regression model examining seasonal and annual variability in monthly pneumonic lung condemnations for three sentinel selection approaches**

**Model**	**IRR**	**95% CI**	**P-value**
**a) Full dataset** ^**1**^			
Winter	---	----	---
Spring	0.97	0.85 – 1.11	0.61
Summer	0.90	0.78 – 1.03	0.12
Fall	0.89	0.78 – 1.03	0.11
2001	---	---	---
2002	0.93	0.79 – 1.08	0.34
2003	0.99	0.85 – 1.16	0.93
2004	0.53	0.44 – 0.63	<0.01
2005	0.45	0.38 – 0.54	<0.01
2006	0.39	0.32 – 0.47	<0.01
2007	0.32	0.26 – 0.40	<0.01
Calves	---	---	---
Cows	1.05	0.87 – 1.27	0.60
Heifers	0.68	0.57 – 0.80	<0.01
Steers	0.75	0.66 – 0.85	<0.01
**b) Abattoirs open all weeks** ^**2**^			
Winter	---	---	---
Spring	1.01	0.88 – 1.16	0.93
Summer	0.92	0.80 – 1.05	0.22
Fall	0.91	0.79 – 1.05	0.22
2001	---	---	---
2002	0.94	0.80 – 1.11	0.50
2003	1.03	0.88 – 1.21	0.68
2004	0.57	0.48 – 0.68	<0.01
2005	0.46	0.38 – 0.55	<0.01
2006	0.40	0.38 – 0.49	<0.01
2007	0.34	0.28 – 0.42	<0.01
Calves	---	---	---
Cows	1.03	0.84 – 1.26	0.75
Heifers	0.73	0.62 – 0.87	<0.01
Steers	0.75	0.66 – 0.86	<0.01
**c) Multi-criteria** ^**3**^			
Winter	---	---	---
Spring	1.08	0.92 – 1.28	0.33
Summer	0.95	0.80 – 1.12	0.51
Fall	0.92	0.78- 1.09	0.35
2001	---	---	---
2002	1.03	0.84 – 1.27	0.76
2003	1.30	1.11 – 1.63	<0.01
2004	1.04	0.85 – 1.28	0.72
2005	0.67	0.54 – 0.84	<0.01
2006	0.67	0.49 – 0.79	<0.01
2007	0.62	0.62 – 0.96	<0.01
Calves	---	---	---
Cows	0.77	0.62 – 0.96	0.02
Heifers	0.62	0.51 – 0.75	<0.01
Steers	0.71	0.61 – 0.82	<0.01
**d) Large abattoirs** ^**4**^			
Winter	---	---	---
Spring	0.97	0.82 – 1.15	0.74
Summer	0.92	0.77 – 1.09	0.32
Fall	0.99	0.84 – 1.18	0.94
2001	---	---	---
2002	1.38	1.11 – 1.72	<0.01
2003	1.56	1.26 – 1.93	<0.01
2004	0.97	0.77 – 1.22	0.78
2005	0.75	0.59 – 0.94	0.01
2006	0.64	0.50 – 0.82	<0.01
2007	0.64	0.50 – 0.82	<0.01
Calves	---	---	---
Cows	1.22	0.92 – 1.62	0.16
Heifers	1.02	0.83 – 1.25	0.86
Steers	0.87	0.74 – 1.03	0.10

^1^Abattoirs processing at least 1 bovine carcass each year of study period (2001 – 2007).

^2^Abattoirs processing at least 1 bovine carcass 52 weeks per year.

^3^Selection of abattoirs meeting the following criteria: processed at least 499 cattle per year, processed at least 1 bovine carcass 44 weeks or more per year, processed cattle representing all animal classes (calves, cows, heifers and steers).

^4^Abattoirs processing ≥ 6500 cattle per year.

## Discussion

Three sentinel site selection approaches for a sentinel syndromic surveillance system using abattoir condemnation data were proposed and compared to the full set of provincially inspected abattoirs in Ontario from 2001–2007. While pneumonic lung condemnation data were used as an exemplar in this study, the process of sentinel abattoir selection could be extended to other disease categories as well. While all selection approaches tended to overestimate the condemnation rates of the full dataset to some degree, the abattoirs open all weeks selection approach appeared to best capture the overall seasonal and temporal trends of the full dataset and would be the most suitable approach for sentinel abattoir selection. This selection approach utilizes data from 45 abattoirs. It may be advantageous to conduct enhanced surveillance at carefully selected sentinel abattoirs rather than at the approximately 100 abattoirs slaughtering cattle in Ontario [14]. This allows for more intensive and specialized training of inspectors for syndromic surveillance. Furthermore, if sentinel abattoirs were selected properly, sentinel abattoirs could help to reduce the cost of surveillance while still being representative of Ontario provincial abattoirs.

While there was not one design approach that perfectly fit the full dataset in all analyses, the results of the descriptive and quantitative analyses found a fairly good fit between abattoirs open all weeks and the full dataset. This sentinel selection approach utilizes less than half of the abattoirs from the full set of abattoirs. By collecting data from fewer abattoirs, the cost of data collection and analysis is reduced. This approach also allows for the use of targeted training of inspectors to reduce any presence of inspector bias on the data, which appeared to be a possible issue with these data based on previous studies [9,10]. The multi-criteria sentinel selection approach also utilizes data from approximately the same number of abattoirs as the abattoirs open all weeks, however, this approach had larger amount of overestimation for heifers and steers than abattoirs open all weeks and did not approximate the overall seasonal, secular and animal class condemnation trends of the full dataset as well as the abattoirs open all weeks. This suggests that the number of abattoirs selected is not necessarily as important as the criteria used to select the abattoirs. While the abattoirs open all weeks sentinel selection approach drastically reduced the number of abattoirs needed to conduct syndromic surveillance involving provincial abattoir condemnation data compared to the full dataset, it is uncertain whether this reduction is sufficient and manageable to conduct intensive and targeted surveillance. Further research is needed to determine if there are other sentinel selection approaches that could reduce this number of abattoirs even further.

Geographical representativeness is also an important consideration when establishing sentinel selection criteria. While the sentinel selection approaches based on abattoirs that are open all weeks and multi-criteria are geographically representative, the large abattoir selection approach is geographically over-representative of abattoirs in central and southern Ontario and has no representation of abattoirs in northern and eastern Ontario. This could pose an issue for syndromic surveillance, as abattoirs generally receive animals from relatively local farms [9], and a lack of representation from abattoirs in these regions could lead to the inability to identify emerging health issues in these under-represented areas. The distribution of abattoirs among regions is almost identical for abattoirs open all weeks selection approach and the full dataset. In addition, region has been shown to be an important variable associated with partial and whole carcass condemnations [8,9] and excluding certain regions could bias the results of spatio-temporal cluster detection methods for syndromic surveillance involving these data.

While the number of abattoirs processing cattle varied over the study period, these fluctuations are likely due to the large economic and regulatory changes in the cattle industry during the study period. If the number of abattoirs fluctuates greatly over time, the process of sentinel selection would have to be repeated on a regular basis and could pose inefficiency issues for the surveillance system. To address this issue in our study, we opted to select only the abattoirs that were open during the study period to represent the full dataset. However, in recent years that number of abattoirs processing cattle has remained fairly consistent and this is not expected to be an issue for sentinel selection in Ontario provincial abattoirs.

This study found bovine provincial abattoir condemnation data to be suitable for sentinel surveillance assuming it is a cost-effective option. However, this study did not investigate outbreak detection, as there were no documented animal health outbreaks in cattle during the study period. Consequently, further research is needed to assess the effectiveness of sentinel selection approaches for detecting emerging health issues using documented or simulated outbreak data of both respiratory diseases as well as other disease syndromes important to both animal health and food safety.

## Conclusions

Sentinel abattoirs show promise for integration into a food animal syndromic surveillance system using Ontario provincial abattoir condemnation data. The selection of the sentinel abattoirs is extremely important in order to maintain representativeness of the disease trends in the entire population. The information extracted from surveillance systems varied by its respective selection approach and for each animal class. While all selection approaches tended to overestimate the condemnation rates of the full dataset to some degree, the abattoirs open all weeks selection approach appeared to best capture the overall seasonal and temporal respiratory condemnation trends of the full dataset and would be most suitable approach for sentinel abattoir selection involving these data. However, should the purpose of the surveillance system be to measure the overall condemnation rates, the overestimation of all sentinel abattoir selection approaches would give inaccurate results. Further studies should examine the performance of the proposed sentinel selection approaches using simulated data that include disease outbreaks for both respiratory diseases as well as other condemnation syndromes relevant to animal health and food safety.
